# Integration Approaches to Model Bioreactor Hydrodynamics and Cellular Kinetics for Advancing Bioprocess Optimisation

**DOI:** 10.3390/bioengineering11060546

**Published:** 2024-05-27

**Authors:** Vishal Kumar Singh, Ioscani Jiménez del Val, Jarka Glassey, Fatemeh Kavousi

**Affiliations:** 1 Process and Chemical Engineering, School of Engineering and Architecture, University College Cork, T12 K8AF Cork, Ireland; jarka.glassey@newcastle.ac.uk; 2 School of Chemical & Bioprocess Engineering, University College Dublin, D04 V1W8 Dublin, Ireland; ioscani.jimenezdelval@ucd.ie; 3 School of Engineering, Newcastle University, Newcastle upon Tyne NE1 7RU, UK

**Keywords:** computational fluid dynamics, process optimisation, cell reaction kinetics, digitalisation, bioprocess modelling

## Abstract

Large-scale bioprocesses are increasing globally to cater to the larger market demands for biological products. As fermenter volumes increase, the efficiency of mixing decreases, and environmental gradients become more pronounced compared to smaller scales. Consequently, the cells experience gradients in process parameters, which in turn affects the efficiency and profitability of the process. Computational fluid dynamics (CFD) simulations are being widely embraced for their ability to simulate bioprocess performance, facilitate bioprocess upscaling, downsizing, and process optimisation. Recently, CFD approaches have been integrated with dynamic Cell reaction kinetic (CRK) modelling to generate valuable information about the cellular response to fluctuating hydrodynamic parameters inside large production processes. Such coupled approaches have the potential to facilitate informed decision-making in intelligent biomanufacturing, aligning with the principles of “Industry 4.0” concerning digitalisation and automation. In this review, we discuss the benefits of utilising integrated CFD-CRK models and the different approaches to integrating CFD-based bioreactor hydrodynamic models with cellular kinetic models. We also highlight the suitability of different coupling approaches for bioprocess modelling in the purview of associated computational loads.

## 1. Introduction

The global market share of biopharmaceutical products is valued at USD 516.79 billion in the year 2024 and is expected to grow at a compound annual growth rate (CAGR) of 8.07% to reach USD 761.80 billion by 2029 [1]. It accounted for 27% of the global pharmaceutical market in 2020, up from 20% in 2015, and is expected to grow faster than the overall pharmaceutical market, with biopharmaceuticals projected to account for 32% of the global pharmaceutical market by 2026 [2]. The high sales growth of biologics has necessitated an increase in the production scale and efforts to achieve higher productivity, efficiency, and cost-effectiveness. However, the transition to large-scale production is always complicated due to the fluctuations in the cell’s environment and bioreactor heterogeneity [3]. Maintenance of homogeneous culture conditions throughout bioreactor volume and batch duration at large scale is often infeasible due to the high energy requirement for the mechanical operation of these bioreactors [4]. This spatial and temporal variation of physiochemical environmental parameters like substrate gradients [5,6,7], dissolved oxygen gradients [8,9,10], and pH gradients [11,12] disturb the physiological state of cells and their cellular machinery. These gradients significantly impact the metabolic response of the cell and culture performance as the cells are subjected to an incessantly fluctuating environment [13]. The heterogeneous process parameter profile in the bioreactor also causes cell population heterogeneity [14].

Both positive and negative effects linked to the presence of environmental gradients have been documented. Outcomes, such as increased cell viability in the presence of glucose gradients [5,6], lower N-glycolylneuraminic acid derivatives at high partial pressure due to carbon dioxide (pCO_2_) accumulation due to mixing gradients [15] and increased specific antibody production at higher culture osmolality and pCO_2_ concentration [16] has been reported in the past. Prior evaluation of the effects of gradients on cellular behaviour can offer insights into upcoming difficulties at a large scale. Countermeasures can be taken in advance to avoid a negative impact on process performance. The expression host’s response to physiochemical gradients can be used to design more robust strains [17]. Traditionally, an experimental approach to address the challenge has been adopted, like mimicking the gradients in process parameters at the lab scale to evaluate scale-up effects [18]. Such scale-down bioreactors present limitations, particularly in their inability to decouple various process parameter gradients, such as dissolved oxygen (DO) and glucose concentrations or pH and osmolarity. Additionally, the frequency and amplitude of environmental changes are heavily reliant on the tested parameter and the specific cell line under examination [19]. The effectiveness of scale-down experiments is significantly influenced by the chosen configuration as the use of geometrically disparate scale-down bioreactors would pose challenges in mimicking the extent and distribution of large-scale gradients. The use of a shake flask and microscale bioreactors as scale-down simulators can potentially lead to conflicting interpretations as they exhibit different oxygen gradients and mass transfer patterns compared to large-scale bioreactors. This impacts cellular physiology, gene expression, and product quality [20]. Moreover, information about the local intensity gradients at large-scale bioreactors is lacking because the addition of multiple probes to collect the data would be intrusive to the fluid flow, and positioning of multiple sampling ports for offline analysis would increase the risk of contamination due to frequent interventions. For these reasons, the design space for scale-down simulators relies heavily on a trial-and-error approach. Real-time experimental characterisation of physiochemical gradients in a turbulent multiphase cell culture fermentation process is complicated, given the system’s complexity, which is influenced by impeller motion, gaseous sparging, feed interventions, foaming-induced mass transfer changes, external heat transfer, and rheological effects [19].

Process modelling techniques like Computational fluid dynamics (CFD) and cell reaction kinetic (CRK) models allow us to predict the occurrence of gradients and assessment of their effect on cellular metabolic behaviour, respectively. CFD facilitates cost and time-effective prediction of the bioreactor hydrodynamics and fluid mixing [21,22,23], mass transfer [24,25], shear stress zones [26,27], and dissolved oxygen profile [27]. Even with the significant success of CFD in bioprocess design and optimisation, the inclusion of all aspects prevalent inside a bioreactor in a simulation space often leads to complicated models. Moreover, their experimental validation is even more challenging [28]. This calls for simplification steps in simulating bioreactor hydrodynamic behaviour. Such simplification mandates prior knowledge and force balance analysis to reveal acting forces with a low magnitude that can be neglected to approach realistic approximations. The trade-off between the use of a simplified model in place of more realistic ones is based upon inherent physical phenomena, the required level of detail, and an acceptable range of errors during validation. Studies utilising CFD as the sole means of bioprocess investigation have only made an indicative impact on process improvement by unravelling the presence of non-ideal behaviour in flow parameters and have aided bioreactor redesign [29,30] as well as process scale-up [31] and scale down [32]. The knowledge gained has led to the identification of hydrodynamic causes of yield loss, but process improvement is still left to rigorous experimentation, as full use of the extensive data generated by CFD cannot be achieved if it is unable to capture cellular responses to spatiotemporal flow information.

On the other hand, standalone cellular kinetic models are unable to predict the complete picture of biological processes as the exclusion of dynamic bioreactor hydrodynamics amounts to confounding of different causes of cell death [33]. Consequently, standalone CRK models do not possess satisfactory predictive capabilities and demand feedback from the surrounding environment to which cells are exposed during the cell culture process [34]. Without this feedback, as is the case with the existing kinetic models, their predictions do not account for scale and its implications, whether a cell culture process is run in shake flasks or in bioreactors of any scale. These models consider all causes of cell death using one parameter, e.g., growth rate. Hence, the performance of the CRK models deteriorates when the model is applied outside the calibration range or when the process conditions change, as the kinetic parameters are highly dependent on the cell line, culture medium, and other bioreactor conditions [35].

Recently, CFD models have been integrated with dynamic CRK modelling to generate valuable information about the cellular response to fluctuating hydrodynamic parameters inside large production processes [36,37]. With the advancement in computing capabilities, including a multitude of factors affecting cellular production has become possible, and bioreactor modelling studies have gradually shifted towards interlinking structured cellular models with CFD simulations using either Eulerian or Lagrangian approaches. In the Lagrangian approach, dispersed entities such as particles, droplets, and bubbles are represented by virtual particles. These virtual particles have tracked properties like positions and velocities, while the continuous phase is represented by a field. This method differs from the Eulerian approach, which utilises concentration and velocity fields to monitor the concentration of dispersed entities. The Eulerian approach is computationally less expensive and more suited for transient simulations. The integration of CFD-CRK for transient analysis of the system has the potential to provide real-time and predictive insights into the dynamic cellular responses, enabling quick decision-making in biomanufacturing processes [38]. It also allows for the creation of digital twins (virtual representations of the bioprocess), enabling simulation and prediction of the impact of various parameters on cellular behaviour. The digitalised approach enhances the efficiency of experimentation, reduces the need for extensive physical trials, and accelerates the development and optimisation of bioproduction processes. Such coupled approaches have the potential to facilitate informed decision-making in intelligent biomanufacturing, aligning with the principles of “Industry 4.0” concerning digitalisation and automation [39].

There have been valuable contributions made in the literature for the application of CFD to model volumetric mass transfer coefficient (k_L_a) [40,41,42], power density (P/V) [43,44,45], mixing time [46,47,48], Kolmogorov length (*λ_k_*) [49,50], shear stress (τ) [51,52,53] and sedimentation [54,55] in bioreactors. Cell culture dynamics, metabolism and product glycosylation have been modelled in the past using mechanistic models [56,57,58], statistical models [59,60,61], and hybrid models [61,62]. The application of CFD for hydrodynamic characterisation and scale-up and CRK for cell metabolism modelling has been extensively reviewed recently [63,64,65,66]. In this review, the focus is on the research articles wherein both the CFD and CRK modelling approaches have been integrated as this strategy has the potential to optimise the whole bioprocess rather than optimisation advances in just unit steps involved in bioprocessing. Special emphasis has been given to the methodology used to integrate the CFD and CRK models along with the process information extracted from such coupling. The literature search methodology has been described in Section 2. A brief about Eulerian and Lagrangian methods and their application in the literature is discussed in Section 3 followed by a discussion on the suitability of these methods for different use cases in Section 4. The state-of-the-art in CFD-CRK literature has been summarised in Section 5. Ultimately, a case has been presented in favour of the coupled CFD-CRK techniques with the rationale of using the benefits of both while being able to cut down on their respective downsides (Section 6).

## 2. Literature Search Methodology

The article selection in this study adheres to the structure outlined by the PRISMA framework [67]. This framework encompasses four key phases: identification, screening, eligibility, and inclusion. The search was executed in April 2024 using the Scopus database. This database was chosen due to its demonstrated ability to effectively retrieve data and ensure the replicability of searches [68]. A total of 77 articles were identified in the literature search based on search criteria mentioned in Table 1. The articles were further screened using Microsoft Excel to curate the final list of eligible articles (26 research articles) to be included for evaluation. Only articles coupling biological reactions relevant to biotherapeutics with CFD have been identified as eligible to match the scope of the current study.

## 3. CFD-CRK Coupling Approaches

### 3.1. Eulerian Approach

This method is also referred to as the Euler–Euler (EE) approach, as the fluid and particulate phases (cells and/or gas bubbles) are not individually tracked. In this approach, all the phases (including the biophase) are treated mathematically as interpenetrating continuums (continuous systems where erratic changes do not occur) and are described in terms of their volume fractions [69]. The assumption of a biological entity as a continuum is an oversimplification and does not consider the individual nature of the cell factory, particularly in the case of unstructured cellular models being used as a counterpart in the coupling process for large-scale bioreactors with longer mixing times. Thus, this methodology is unable to account for the individual response of the cells [70].

In order to consider cells as individual entities, Population Balance Models (PBMs) can be used, which can account for the population adaption dynamics of cells [71,72]. Rather than assuming equilibrium between internal and external state parameters of cells, PBMs allow for capturing local non-equilibria by using a distribution function for intrinsic cellular parameters based on the principle of segregation. Most studies have used specific growth rates as the representative parameter to differentiate between individual cells [72,73]. By using PBMs for an *Escherichia coli* fermentation process, glucose gradient-induced acetate overflow zones were identified [72]. The biggest advantage of using this approach is its scalability to large domains [70]. This approach has also been demonstrated viable to model cell culture processes for the entire batch duration (assuming pseudo-steady state hydrodynamics) and in the identification of decreased process yield causes for macro-mixed bioreactors [71]. Most studies assume one-dimensional heterogeneity (captured by specific growth rate), which may be insufficient in cases where the adaptation time for cells is much greater than the reaction time, as this will require resolution of heterogeneity across two-time scales. One limitation of PBM is the inclusion of high-dimension functions when combined with structured kinetic models, which is computationally expensive. Also, the particle (cells) travel history inside the fluid domain cannot be accounted for.

### 3.2. Lagrangian Approach

The Lagrangian approach emphasises tracking the fate of each particle individually. Consequently, this approach results in a substantial number of ordinary differential equations to be solved and, therefore, is highly computationally intensive. Due to computational expense, Euler-Lagrange (EL) simulations with Eulerian reaction coupling were initially adopted to model the extracellular environment of the cells but failed to describe the interaction of extracellular and intracellular culture phenomena [74,75].

A more practical approach to model fluid dynamics and cellular physiology interactions is to apply the Lagrangian view to the cells while using an Eulerian framework for fluid dynamics. This modelling strategy is called Euler–Lagrange simulations with Lagrangian reaction coupling or agent-based methodology [76]. In this case, the biotic phase is viewed as clusters of cells and is represented by computational particles (parcels). To describe cellular metabolism, a structured kinetic model is formulated by lumping important intracellular metabolites and enzymes in different pools. Within each parcel, the intracellular composition is tracked by assigning a composition vector for each pool using the Lagrangian approach. In this way, each pool is quantified by a single value for the defined composition vector, making the pool interactions straightforward. However, unlike PBM, there are no pseudo-steady states for the Lagrangian phase, requiring a transient solution with short timesteps to capture parcel motion and pool dynamics. A 9-pool metabolic model characterised by five metabolite pools: glycolytic intermediates, amino acids, Adenosine triphosphate, Phenylacetic acid, and stored carbohydrates; and four pathway enzyme pools (glucose uptake, Phenylacetic acid export, penicillin production, and storage conversion) for *Penicillium chrysogenum*, showed sufficient accuracy in predicting extracellular concentrations and reaction rates [77].

The first application of this approach was the study of temporal oscillations in glycolysis pathway metabolites in the presence of heterogenous glucose concentrations at the single-cell level for *Saccharomyces cerevisiae* cells [78]. The authors acknowledged the computational burden of three-dimensional discretisation and the inclusion of a sufficient number of cells in each control volume in the modelling step. Hence, the agent-based modelling method can accommodate intracellular pools and structured cellular kinetic models, but it is not computationally tractable to combine spatially resolved transient multiphase bioreactor models with structured segregated cellular kinetic models [78].

Since the Lagrangian approach is computationally intensive, appropriate approximations are mandated in both the CFD modelling as well as in the metabolic models. However, the argument of using lumped cellular models to reduce the computational cost is challenging to implement, as it is difficult to determine the number of particulates (cells) required to avoid gradients in cell concentration [77]. If local variations in metabolite consumption rates are to be included, the required number of particles will increase. An alternative approach is to compartmentalise the CFD computational domain. This strategy has offered good results in syngas fermentation applications [79] and avoiding non-essential details in the model based on prior knowledge can yield better simulation results [80]. Table 2 provides a comparison of the non-compartmentalised CFD modelling approaches to the compartmentalisation-based modelling approach [81].

## 4. Selecting a Suitable Coupling Strategy

When there is no limitation on computational cost, the Lagrangian description of a system is the most potent method and provides a more detailed description of the process. However, the order of particles that need to be tracked can range upwards of 10^6^ (especially in CHO cell culture processes [82]). Hence, it becomes a necessity to rationally select the coupling strategy as the simulation can become infeasible otherwise. The decision to opt for full Lagrangian coupling depends on how the metabolic (uptake) reactions are affected by the intracellular activities. If the effect of the intracellular state of a cell on the extracellular reactions is negligible, also referred to as one-way coupling, then the intracellular environment can be assumed to be homogeneous, and the Eulerian approach will be able to provide satisfactory results. If there is a heterogenous intracellular environment, uptake rates will be affected (two-phase coupling or interphase coupling), and, hence, the fate of each particle is expected to affect the process dynamics [77]. Particle loading and Stokes number can be used as derived parameters to serve as guiding factors to select whether a phase (fluid/particles/cells) should be tracked using the Lagrangian method [76], as detailed below.

Most bioreactors operate with at least 1% (percentage by volume, *v*/*v*) gas (which is equivalent to an L/d_p_ value of 3.74—still considerably less than high gas loading for which the L/d_p_ value is 8) [76]. This indicates that the gas loading is at least an intermediate parameter (i.e., not low) in nature, increasing two-way interphase coupling between the gas and liquid (i.e., the bubbles also influence the carrier liquid via a reduction in mean momentum and turbulence). So, the Lagrangian approach should be applied to gas bubbles here. Similarly, a 1 × 10^6^ cells/mL suspension of CHO cells with an approximate cell diameter of 15 μm equates to a volume fraction (percentage by volume, *v*/*v*) of 0.2% and a corresponding L/d_p_ value of 6.7, suggesting an intermediate particle loading for which the Lagrangian approach is required [76]. Thus, Euler–Lagrange simulations with Lagrangian reaction coupling should be adopted. This decision is further supported by the Stokes number (St), which is defined as the ratio of particle response time to system response time (Equation (1)).

(1) $$St=\frac{\left[\frac{\left(\rho -\rho^{\prime }\right){d_{p}}^{2}}{18}µ\right]}{\left(\frac{L}{v}\right)},$$

where *ρ* is the density of the liquid, *ρ*′ is the density of the dispersed or particulate phase, *d_p_* is the particle diameter, µ is the dynamic viscosity of the liquid, *L* is the characteristic system length, and *v* is the characteristic velocity.

**Figure 1:**
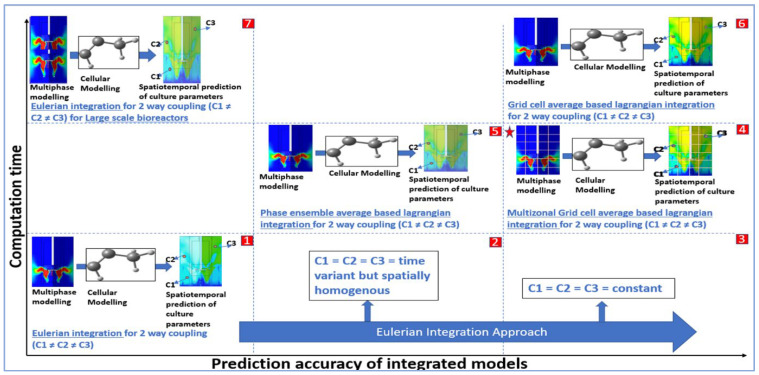
Computation time vs. Prediction accuracy for small scale cell culture process (except (7)) (1) Eulerian integration for spatiotemporally heterogeneous bio-phase, i.e., cellular parameters at position 1 (C1), position 2 (C2) and position 3 (C3) are different during bioproduction phase, requires low computational cost but results in low prediction accuracy (2) Prediction accuracy of Eulerian approach increases for temporally heterogenous but spatially homogenous systems (3) Eulerian approach is best suited for homogeneous cellular behaviour offering high prediction accuracy while maintaining low computational cost (4) Multizonal compartmentation based Lagrangian integration is faster and feasible while offering comparable model accuracy to full Lagrangian coupling (5) Phase ensemble average based Lagrangian integration for heterogeneous bio-phase is able to accommodate the time variance in cellular states via averaging but cannot capture the spatial heterogeneity associated with the cells (6) Full Lagrangian coupling for heterogeneous systems with particle (cell) tracking yields high prediction accuracy of optimising parameters but is computationally intensive and suited for reduced and stable cellular kinetic models (7) Large scale bioreactors require high computational time for the fluid phase, which makes the integration of the bio-kinetic phase unfeasible and awaits technology enhancement to prove its utility. Note: The post-processed simulation results are for representative purposes and do not indicate the exact distribution of parameters.

If the characteristic length for a stirred-tank bioreactor is estimated as D_i_ (the impeller diameter), the characteristic velocity as ND_i_ (the tip speed), a bubble size of 5 mm (corresponds to $\frac{\left(\rho -\rho^{\prime }\right)d^{2}}{18}µ$ = 1.3), and for an agitation rate of 50 rpm ($\frac{L}{v}$ = 1 s), St is estimated to be 1.3 [76], which favours the use of the Lagrangian approach because particles will move independently of the fluid flow at Stokes number greater than 1 [83]. Particles with a Stokes number greater than one do not follow the fluid streamline as they are dominated by their inertia and tend to continue along their initial trajectory [84]. Figure 1 depicts a performance evaluation diagram for building a CFD-CRK coupled model, which can be computationally feasible and serve optimum prediction accuracy to make informed decisions regarding cell culture process development. The suitability of a particular coupling strategy, graphically represented in Figure 1, is explained in Table 3.

## 5. State of the Art

The foundation of CFD-CRK coupling to achieve additional process information was laid with the integration of CFD models and unstructured kinetic models to describe fed-batch *Saccharomyces cerevisiae* culture [85]. The authors attempted to demonstrate the presence of glucose gradients within the bioreactor (total volume 30 m^3^) and found that the nature of gradients varied with the location of feeding points: more homogenous glucose concentration was observed in the bottom fed process than in the top fed one. Deviations across time and position were observed between simulated and measured glucose concentrations, in particular at higher cell densities. These discrepancies could have been due to errors in kinetic parameters for glucose balance (e.g., the yield coefficient of cells on glucose) or lower axial mixing predicted with the CFD model [85]. Although this type of integrated model was able to offer insights into bioreactor hydrodynamics, it failed to decipher the response of cells to such conditions because the adaptation of cellular physiology to the bioreactor surroundings was not included [85]. The same problem persisted when simplistic empirical models were used to correlate the Reynolds number to the integral viable cell density of CHO-320 cells producing interferon-γ in a stirred tank bioreactor [86]. Consequently, no unifying correlation could be formulated to capture the physiological response to the changing bioreactor hydrodynamics.

Recently, Nadal-Rey et al. [87], the particle lifeline methodology was employed to assess the impact of reactor design on the conditions encountered by two frequently utilised industrial microorganisms, *Escherichia coli* and *Saccharomyces cerevisiae*. The findings revealed that cells in the stirred tank reactor were more likely to undergo extended periods of both starvation and overflow metabolism compared to those in the bubble column. This pattern was attributed to the differences in mixing characteristics between the two reactor designs. Remarkably, a substantial portion (60%) of the population in the stirred tank reactors was found to be in starvation conditions for a majority of the time (>70%), a situation that could potentially influence cellular metabolism. In a similar study [36], the authors evaluated the effect of carbon monoxide gradients on *Clostridium ljungdahlii* in a 125 m^3^ bubble column bioreactor. It was found that 97% of cells faced substrate limitations, while 84% were prone to transcriptional changes after prolonged exposure to stress-inducing conditions (over 70 s). Bacterial movement primarily occurred between regions of low and moderate product biomass yield, with longer residence times in the latter. The circulation time, determined through mixing time analysis, resembled the average circulation time of a single bacterium. These findings were inferred from regime transition studies, which also identified maximum residence times exceeding 100 s and minimum regime crossing times of 10 s. Since the experimental data for the effect of these stress-inducing conditions on *Clostridium ljungdahlii* was not available, the application of lifeline analysis data was not utilised for optimising the cell culture process. Also, it is difficult to draw an inference obtained from process snapshots which can be generalised for the whole duration of the batch. Such information can be used to design bioprocess vessels, manipulate operating conditions and even adjust bioreactor configuration to optimise the process [87]. The development of scale-down simulators using this approach holds promise for process life-cycle management and process scaling [70].

If the impact of process parameter gradients on cellular behaviour can be predicted for the entire duration of the production process, significant leads can be gained for the development of strategies to enhance productivity [37], increase efficiency [38], and reduce costs [81], contributing to the goals of process intensification. In one of the studies, for optimisation of vanillin production from ferulic acid using recombinant *Escherichia coli* cells, Yeoh et al. [37] evaluated the impact of mass transfer and aeration rate on the process performance. A coupled CFD-CRK model in transient mode was applied for the entire batch duration to optimise the batch process which resulted in a highly optimised bioprocess achieving 94% bioconversion yield. However, due to computational challenges, applying the transient CFD-CRK model for mammalian cell culture fed-batch process or perfusion process is not possible currently [77,87]. A limited number of integrated CFD-CRK models have been developed for mammalian cells [38,88], likely because modelling of their cellular kinetics requires a more detailed description of metabolic reactions which increases the computational cost. Farzan et al. studied the effect of dissolved oxygen (DO) concentration, bubble diameter, and turbulent eddies on the growth, viability and productivity of CHO cells via coupling Ansys Fluent simulated flow information with a biokinetic model using a non-linear solver (details of the solver not specified) [38]. The focus of this study was more on exemplifying the coupling algorithm for CHO cells and the identification of optimal operating conditions. Hence, experimental validation of the proposed optimal operating schemes was not included. Recently, Oliveira et al. successfully applied and experimentally validated an integrated CFD-CRK model to demonstrate optimal PID controller embedding to predict changes in oxygen and pH in the cell culture system [88]. This study was conducted in the GE Xcellerex^TM^ XDR 200 bioreactor using a proprietary mammalian cell line. The authors emphasised the need for advancements required in GPU architecture to apply such a real-time coupling between reactor operating conditions and process outcomes for large-scale bioreactors.

The majority of the studies mentioned in Table 4 have considered unstructured growth models for microbial cell lines with the hydrodynamics solved using commercial CFD software such as Ansys Fluent (https://www.ansys.com/products/fluids/ansys-fluent, accessed on 25 April 2024) [36,38,75], Ansys CFX (https://www.ansys.com/products/fluids/ansys-cfx, accessed on 25 April 2024) [37,87], M-Star CFD (https://mstarcfd.com/, accessed on 25 April 2024) [88,89], COMSOL Multiphysics (https://www.comsol.com/comsol-multiphysics, accessed on 25 April 2024) [90], and PHOENICS (https://www.cham.co.uk/phoenics.php, accessed on 25 April 2024) [78]. It is interesting to note that open-source platforms like OpenFOAM and OpenLB (https://www.openlb.net/, accessed on 25 April 2024) have not been applied yet to integrate kinetic models with CFD models. Most of the studies have used the finite volume method for modelling the bioreactor hydrodynamics that was integrated with mechanistic models describing the culture metabolic dynamics. Since simulation time for transient modelling is currently a challenge, these software support parallel computing using Graphics processing units (GPU) in their latest versions. By default, computational software like COMSOL (https://www.comsol.com/, accessed on 25 April 2024), Autodesk CFD (https://www.autodesk.com/products/cfd/overview, accessed on 25 April 2024), Ansys CFX, and OpenFOAM (https://www.openfoam.com/, accessed on 25 April 2024) are not configured to utilise GPUs for calculations. However, there exist several modifications, one of which is MixIT (https://tridiagonal.com/mixit/, accessed on 25 April 2024), that enable OpenFOAM to leverage the computational power of GPUs [91].

Validation of the CFD-CRK model results with experimental data is critical to assess the extent to which a model accurately mirrors reality. However, current technologies in experimental measurement of spatiotemporal variation of process parameters are limited [92]. Most of the studies of the work described in Table 4, have used validation approaches wherein either the hydrodynamic parameters from CFD simulations have been validated using thermal anemometry [85], Laser Doppler Velocimetry (LDV) [86], and Particle Image Velocimetry (PIV) [71] data. The mean concentrations of metabolites and products have been also validated against experimental data acquired from the experiments [37,88,93]. However, only a few studies have used multiple sensors placed at different locations within the bioreactor space to validate the results [85,94]. It is important to acknowledge that the experiments are not exempt from measurement inaccuracies, and these errors must be factored into the statistical analysis especially when multiple sensors are used to extract the same data. A few studies involving detailed kinetic models for cell metabolism have not been available to validate the results and have presented in silico results as proof-of-concept [36,38,75,87,95,96]. Table 4 summarises relevant available year-wise research outputs, studied cell culture system, adopted approaches, and validation strategy.

**Table 4:** Summary of CFD-CRK integrated models in cell culture.

Reference	System Description	Integration Approach	Validation Method	Year
[88]	Proprietary mammalian cell line, 200 L bioreactor	Euler–Lagrange (EL) simulations with Eulerian reaction coupling. The only reaction to model oxygen and pH profile has been coupled	Modelled Oxygen and pH concentrations verified experimentally	2024
[87]	*Escherichia coli* and *Saccharomyces cerevisiae*, 90 m^3^ bubble column and a stirred tank bioreactor	Euler–Euler simulations with Lagrangian particle tracking approach for cells. Unstructured kinetic model for growth and nutrient uptake rates	Results were completely in silico	2023
[90]	*S. cerevisiae*, 22 m^3^ stirred tank reactor	Euler–Lagrange (EL) simulations with Lagrangian reaction coupling. Unstructured model for nutrient uptake rates	Glucose concentration field data validated against data from literature [85]	2023
[95]	Cell line not mentioned, Perfusion bioreactor volume not mentioned	Euler–Euler simulations with unstructured kinetic model for growth and substrate uptake	Results were completely in silico	2022
[93]	*P. chrysogenum*, 54 m^3^ stirred tank reactor	Euler–Lagrange (EL) compartment-based CFD simulations with Lagrangian reaction coupling. Unstructured and structured cellular model for growth rate distribution	The kinetic model was validated against literature data [97], and the CFD-CRK model was validated against mean specific growth rate data from industrial experimental data	2022
[37]	*E. coli*, 1.5 L stirred-tank bioreactor	Euler–Lagrange (EL) simulations with Eulerian reaction coupling. Unstructured cellular model for the effect of dissolved oxygen on cell growth	Volume average of substrate and product concentrations were validated against experimental data	2021
[94]	*Streptococcus thermophilus*, 700 L stirred tank reactor	Euler–Euler simulations with unstructured kinetic model for growth and pH change	pH gradient results generated from the CFD-CRK model were experimentally validated using 6 multiple probes at different locations	2019
[36]	*Clostridium ljungdahlii*, 125 m^3^ bubble column	Euler–Lagrange (EL) simulations with Lagrangian reaction coupling. stoichiometry model for carbon monoxide uptake	Results were completely in silico	2019
[98]	*P. chrysogenum*, 54 m^3^ stirred tank reactor	Euler–Lagrange (EL) simulations with Lagrangian reaction coupling. Structured cellular model for growth rate distribution	The kinetic model was validated against the literature data [97], and the CFD-CRK model was validated against mean specific growth rate data from industrial experimental data	2018
[38]	*CHO cell*, 3 L bioreactor	Euler–Lagrange (EL) simulations with Lagrangian reaction coupling. Unstructured cellular model for the effect of DO on cell growth	Results were completely in silico	2018
[77]	*P. chrysogenum*, 98 m^3^ hypothetical reactor	Euler–Lagrange (EL) simulations with Lagrangian reaction coupling. Unstructured model for nutrient uptake rates	Mean concentration profiles were compared against the literature data	2017
[74]	*S. cerevisiae*, 22 m^3^ stirred tank reactor	Euler–Lagrange (EL) simulations with Lagrangian reaction coupling. Unstructured model for nutrient uptake rates	Glucose concentration field data validated against data from the literature [85]	2017
[75]	*Pseudomonas putida*, 54 m^3^ stirred tank reactor	Euler–Lagrange (EL) simulations with Lagrangian reaction coupling. An unstructured cellular model involving logistic equation for growth	Results were completely in silico	2017
[96]	*HFN 7.1 murine hybridoma cells*, 0.01 m^3^ stirred tank reactor	Euler–Lagrange (EL) simulations with Lagrangian reaction coupling. Unstructured cellular model for the distribution of cells over cell cycle and effect of pH, dissolved oxygen, gas holdup and energy dissipation rate on cell metabolism	Results were completely in silico	2017
[99]	*Carthamus tinctorius* L., 5 L–15 L stirred tank reactor	Euler–Lagrange (EL) simulations with Lagrangian reaction coupling. Unstructured model for death kinetics	CFD results were validated by Particle Image Velocimetry (PIV) measurements, and death kinetics was validated experimentally by viability data.	2016
[100]	*S. cerevisiae*, 0.24 m^3^ bubble column reactor	Euler–Lagrange (EL) simulations with Lagrangian reaction coupling. Unstructured model for nutrient uptake rates	CFD and kinetic models were validated against the literature data [101,102]. No validation of the glucose concentration field.	2016
[70]	*Penicillium chrysogenum*, 54 m^3^ stirred tank reactor	Euler–Lagrange (EL) simulations with Lagrangian reaction coupling. Unstructured model for nutrient uptake rates	Mean substrate concentrations were validated against simulated average substrate concentrations from the Eulerian method.	2016
[72]	*E. coli*, 22 m^3^ stirred tank reactor	Euler–Euler simulations with Population balance models for cells. Structured model for growth rate distribution	The volume average of substrate and product concentrations was validated against experimental data and data from the literature [103].	2015
[71]	Cell line not mentioned, 0.07/70 m^3^ stirred tank reactor	Euler–Euler simulations with Population balance models for cells. Unstructured model for growth rate distribution	Fluid flow validation from Particle Image Velocimetry (PIV) measurements from the literature [104,105]. No validation for the spatial distribution of specific growth rates.	2014
[73]	Hypothetical aerobic bacteria (like Candida tropicalis), 3-L reactor	Euler–Euler simulations with Population balance models for cells. Unstructured model for nutrient uptake rates	Results generated from the CFD-CRK model were validated against the literature data from [106]	2013
[107]	*Aspergillus niger*, 5 dm^3^ stirred-tank bioreactor	Euler–Euler simulations with Eulerian reaction coupling. Unstructured cellular model for growth and product formation	Numerical results are validated against experimental data for a batch time of 60 h	2013
[86]	*CHO 320 producing interferon-γ*, 1.4 L regulated stirred tank reactor	Empirical Methodology: experimental data fitting to correlate hydrodynamic parameters (mean turbulent energy dissipation rate and Reynolds number) to integral viable cell density	The fluid flow field was validated by Laser Doppler Velocimetry (LDV) measurements. Experimental validation of empirical correlation for hydrodynamic parameters to integral viable cell density	2010
[108]	Fibroblast growth factor-2 (FGF-2) producing endothelial cells, FiberCell^®^ hollow-fibre (bioreactor volume not mentioned)	Euler–Euler simulations with Eulerian reaction coupling. Unstructured cellular model for protein production	Concentration of protein till 600 s at the boundary wall of fibre validated against the experimental data	2010
[83]	*Escherichia coli*, 0.9 m^3^ stirred tank reactor	Euler–Lagrange (EL) simulations with Lagrangian reaction coupling. Structured cellular model applied for sugar uptake	CFD simulations and kinetic model validation with experimental data from literature [109,110]. Glucose concentration field data is qualitatively verified from experimental observations from the literature [85].	2006
[78]	*Saccharomyces cerevisiae*, 68 L and 900 L stirred tank reactor	Euler–Lagrange (EL) simulations with Lagrangian reaction coupling. Structured cellular model applied for glycolysis pathway	Lagrange strategy validation by mixing time experiment with tracer substance. No validation for oscillating yeast cells in spacetime	2004
[85]	*Saccharomyces cerevisiae*, 30 m^3^ stirred tank reactor	Euler–Euler simulations with Eulerian reaction coupling. Unstructured cellular model for growth and product formation	Hydrodynamic parameters were validated against experimental data from thermal anemometry. Nutrient concentrations were validated against data collected from 3 sensors at different locations	1996

## 6. Conclusions and Future Prospects

Prediction of cellular responses to external perturbations requires approaches that integrate descriptions of metabolic and hydrodynamic phenomena. Such integrated methods provide useful insights into cellular physiology, cell population dynamics and metabolite consumption/production rates. The structured cellular model can capture the intracellular physiochemical parameters and population heterogeneity prevalent in the process. The combination of CFD and CRK models represents a digitalised approach to understanding and optimising biomanufacturing processes. This digitalised approach enhances the efficiency of experimentation, reduces the need for extensive physical trials, and accelerates the development and optimisation of bioproduction processes. Currently, the experimental validation of these models using multi-compartment reactors consisting of stirred tank reactors and plug flow reactors or by using flow-inhibiting elements is challenging. New technical developments in flow-following sensor particles and microfluidic single-cell cultivation are opening the door to a more interdisciplinary attempt to validate CFD-CRK models and even evaluate process parameter gradient as a time series of position, cellular state and extracellular metabolite concentrations.

However, these approaches present important challenges. Models of cell culture kinetics are often constructed by lumping metabolite pools and often lack descriptions of important intracellular states, such as redox potential and energy charge. In addition, the parameterisation of these models requires repeated cycles of validation via further experimentation. Recent progress in hybrid modelling techniques, where reaction rates are replaced by data-driven approaches, such as partial least squares, artificial neural networks, and Gaussian processes, holds promise for enhanced model performance [111].

From the CFD modelling aspect, especially for long-duration fed-batch and continuous manufacturing processes, computation time/expense is the overall challenge. A way around this problem is the use of compartment modelling, which assumes steady-state conditions for the selected compartments [72,112]. Recurrence-CFD (rCFD) models based on the creation of recurring patterns of dynamic flow [113], reduced order models, GPU-enabled parallel processing, quantum CFD and applications such as ANSYS Discovery Live are opening new horizons to not only realise computational feasibility but also to accommodate rheological characteristics which are closer to the actual fermentation broth. Nevertheless, these developments are bringing exciting times ahead to bring about an efficient modelling framework that can capture the physiological properties of cells, the dynamic physical conditions of the bioreactor, and the crosstalk between these two.

## Figures and Tables

**Table 1:** Search criteria for research article screening on Scopus database.

Database	CFD Modelling		CRK Modelling		System	Limit to
Scopus	“Computational Fluid Dynamics” OR “CFD”	**AND**	“Cell Reaction Kinetics” OR “Kinetic modelling”	**AND**	“Fermentation” OR “Bioreactor” OR “Bioprocess” OR “Bioprocess development”	**Source Type:** Journals **Document Type:** Articles, Conference papers **Language:** English

**Table 2:** Non-compartmentalised CFD modelling approaches vs. compartmentalisation-based approach [81].

Factors	CFD Approach		Compartmentalisation-Based Modelling Approach
	Eulerian Approach	Agent-Based Methodology	
Computational effort	High	High	Low
Level of accuracy	High	High	Low
Prediction accuracy of flow regime	High	High	Low
Single-cell tracking	No	Yes	Yes
Integrable model size	Coarse-grained small-scale	Coarse-grained small-scale	Genome-scale
Number of particles	High	Low (<10%)	High

**Table 3:** Applicability of CFD-CRK coupling strategy for different bioprocess conditions scenarios.

Bioreactor Physical Conditions	CFD-CRK Coupling Strategy	Computational Load	Model Prediction Accuracy	Remarks
Small-scale bioreactor with heterogenous culture parameters in spacetime	Eulerian integration	Low	Low	The application of Eulerian integration to a heterogeneous bioprocessing environment is an oversimplification of the system, which leads to poor prediction of culture parameters [76]. This coupling strategy can be used to integrate complex and unstable cellular models to formulate the sample space for the process conditions to be further evaluated.
Small-scale bioreactor with temporally heterogeneous but spatially homogeneous culture parameters	Eulerian integration	Low	Medium	The assumption of spatially homogeneous culture parameters represents an ideally mixed system, which can be loosely approximated to be the scenario in small-volume bioreactors with ample agitation and aeration. In such cases, model prediction is postulated to increase as cellular behaviour is only temporally impacted.
Small-scale bioreactor with homogeneous culture parameters in spacetime	Eulerian integration	Low	High	The assumption of homogeneous culture parameters in spacetime is a hypothetical case. The closest example of such a case is the production phase of a small-scale, continuously perfused cell culture process, as the nutrient availability and distribution happen in a close to uniform environment. For this duration of steady state, Eulerian coupling can provide higher prediction accuracy.
Small-scale bioreactor with heterogenous culture parameters in spacetime	Phase ensemble average-based Lagrangian integration	Medium	Medium	Phase ensemble average-based Lagrangian integration ignores the spatial heterogeneity of the parameters and uses a time-averaged approach to account for the temporal variation of culture parameters. This increases the computational time as well as the accuracy of the predicted model compared to the Eulerian approach, which completely ignores the presence of heterogeneous culture parameters in spacetime.
Small-scale bioreactor with heterogenous culture parameters in spacetime	Grid cell average-based Lagrangian	High	High	Grid cell average-based Lagrangian approach tracks the cells for the variations in cell behaviour in spacetime. Clearly, this approach is computationally expensive but offers high-quality resolution in terms of prediction accuracy. Such a coupling approach can be applied to small-scale bioreactors with reduced cellular models.
Small-scale bioreactor with heterogenous culture parameters in spacetime	Multizonal grid cell average-based Lagrangian	Medium to High	Medium to High	The multizonal grid cell average-based Lagrangian approach divides the bioreactor 3D space into multiple compartments and assumes spatial uniformity within these zones. This approach reduces the computational burden as compared to the non-compartmentalised grid cell average-based Lagrangian approach, and the appropriate selection of multizone ensures medium to high prediction accuracy.
Large-scale bioreactor with heterogenous culture parameters in spacetime	Eulerian integration	Low	Low	For large-scale bioreactors, CFD models are less computationally tractable, thereby increasing the simulation time [78]. Eulerian coupling is the only feasible approach currently, and technological advancements in computing are required to move to better coupling approaches capable of providing more realistic prediction accuracy.

## Nomenclature

Abbreviations	
USD	United States Dollar
CFD	Computational fluid dynamics
CRK	Cell reaction kinetic
DO	Dissolved oxygen
EE	Euler-Euler
EL	Euler-Lagrange
LDV	Laser Doppler Velocimetry
PBMs	Population balance models
PIV	Particle Image Velocimetry
rCFD	Recurrence-CFD
TVD	Total Variation Diminishing
Symbols	
µ	Viscosity of the liquid (cP)
Di	Impeller diameter (m)
*d_p_*	Particle diameter (m)
k_L_a	Volumetric mass transfer coefficient (1/h)
*L*	Distance between particles/characteristic system length (m)
N	Agitation rate
P/V	Power density (W/m^3^)
St	Stokes number
*v*	Characteristic velocity (m/s)
*λ_k_*	Kolmogorov length (μm)
*ρ*	Density of liquid (kg/m^3^)
*ρ*′	Density of dispersed or particulate phase (kg/m^3^)
τ	Shear stress (N/m^2^)
